# Ferulic Acid as an Anti-Inflammatory Agent: Insights into Molecular Mechanisms, Pharmacokinetics and Applications

**DOI:** 10.3390/ph18060912

**Published:** 2025-06-18

**Authors:** Jiaying Liu, Yu Guan, Le Yang, Heng Fang, Hui Sun, Ye Sun, Guangli Yan, Ling Kong, Xijun Wang

**Affiliations:** 1 State Key Laboratory of Integration and Innovation of Classic Formula and Modern Chinese Medicine, National Chinmedomics Research Center, National TCM Key Laboratory of Serum Pharmacochemistry, Metabolomics Laboratory, Department of Pharmaceutical Analysis, Heilongjiang University of Chinese Medicine, Heping Road 24, Harbin 150040, China; 2 State Key Laboratory of Dampness Syndrome of Chinese Medicine, The Second Affiliated Hospital Guangzhou University of Chinese Medicine, Dade Road 111, Guangzhou 510000, China

**Keywords:** ferulic acid, anti-inflammatory mechanism, inflammatory diseases, pharmacokinetics, cytotoxicity, clinical translation

## Abstract

Ferulic acid (FA), a hydroxycinnamic acid derivative, is a key bioactive component in traditional medicinal plants including *Angelica sinensis* and *Asafoetida*. Accumulating evidence supports its therapeutic efficacy in inflammatory disorders, such as rheumatoid arthritis (RA) and ulcerative colitis (UC). FA exerts anti-inflammatory effects through (1) the regulation of inflammatory cytokine levels; (2) modulation of signaling pathways such as nuclear factor kappa B (NF-κB), mitogen-activated protein kinase (MAPK), and janus kinase/signal transducer and activator of transcription (JAK/STAT); (3) amelioration of oxidative stress; and (4) regulation of immune cell homeostasis. At the pharmacokinetic level, studies show that FA is rapidly absorbed but exhibits low bioavailability, mainly due to the influence of metabolic pathways and food matrix characteristics. This review systematically summarizes the literature on the anti-inflammatory effects of FA, covering molecular mechanisms, pharmacokinetic characteristics, and application scenarios. Preclinical studies show that FA has low toxicity and good safety, demonstrating potential for development as a novel anti-inflammatory drug. However, its clinical translation is hindered by bottlenecks such as low bioavailability and insufficient human clinical data. Future research should prioritize developing novel drug delivery systems and conducting large-scale clinical trials to facilitate its clinical translation.

## 1. Introduction

Inflammation is a defense mechanism triggered by chemical, mechanical, or microbial injuries, aiming to eliminate the injury source and restore tissue function [1], and is generally regarded as a self-limiting and beneficial healing process [2]. As early as over 2000 years ago, the cardinal signs of inflammation—redness, swelling, heat, and pain—were described [3,4]. Inflammation is categorized into acute and chronic types based on its duration. Acute inflammatory reactions (e.g., surgical trauma) are self-limiting [5], while chronic inflammation is usually associated with age-related chronic diseases such as cardiovascular, neurodegenerative, and metabolic disorders (e.g., type 2 diabetes) [1,2]. However, current anti-inflammatory drugs, encompassing nonsteroidal anti-inflammatory drugs (NSAIDs) and glucocorticoids, are associated with significant side effects [6]. As a result, the search for safer and more effective therapeutic approaches has emerged as a critical research priority.

**Figure 1:**
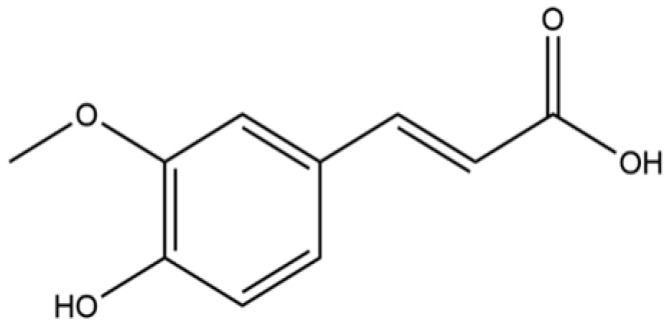
Chemical structure of FA.

In recent years, a growing number of researchers have shifted their focus on treating inflammatory diseases to the study of traditional Chinese medicines (TCMs) and their components. For example, baicalin and andrographolide can attenuate inflammatory responses by inhibiting signaling pathways such as NF-κB [7,8]. Similarly, FA emerges as a promising anti-inflammatory candidate. It exerts effects via multiple mechanisms, including the modulation of NF-κB, MAPK, and JAK/STAT, and the suppression of key proinflammatory cytokines [tumor necrosis factor-α (TNF-α), interleukin-1β (IL-1β), and IL-6], with these mechanisms collectively attenuating inflammatory pathology. Chemically named 4-hydroxy-3-methoxycinnamic acid (C_10_H_10_O_4_, MW 194.184), FA exists as cis/trans isomers (pale yellow solids; Figure 1). It is abundant in Apiaceae plants (e.g., *Angelica sinensis* and *Asafoetida*), serving as a key active component [9,10]. Studies have shown that FA exhibits diverse pharmacological characteristics, such as anti-inflammation, antioxidant, and antitumor [11,12]. Among these, its anti-inflammatory potential has received increasing academic attention [13]. In 2000, Akihisa T. et al. discovered that FA salt exhibited anti-inflammatory effects in mice [14], with subsequent studies confirming its activity [15,16,17]. Recently, FA has shown remarkable efficacy in treating inflammation in neurological, cardiac, respiratory, and digestive disorders, as well as osteoarthropathies. Alongside its anti-inflammatory effects, FA’s pharmacokinetics have been extensively studied in vivo, which underpins its anti-inflammatory mechanisms.

This review summarizes research from the past decade on the anti-inflammatory mechanisms, pharmacokinetics, toxicity, and applications of FA, based on a literature search using keywords such as “FA”, “inflammation”, “pharmacokinetics”, and “toxicity” in databases including PubMed, Google Scholar, and Science Direct. The key anti-inflammatory mechanisms of FA across diseases and models are summarized in Table 1, highlighting targeted pathways and outcomes. This review hopes to provide researchers with a more detailed and comprehensive reference for using FA in the treatment of inflammatory diseases. Overall, these results indicate that FA demonstrates promise as a therapeutic candidate for inflammatory diseases.

## 2. Anti-Inflammatory Pharmacological Properties of Ferulic Acid

Modern pharmacological studies confirm that FA exhibits significant anti-inflammatory activity. Its mechanisms primarily involve the modulation of inflammation-related signaling pathways, inhibition of oxidative stress, regulation of cell adhesion molecule (CAM) expression, and immunomodulation effects. The following section focuses on elucidating its regulatory roles in these signaling pathways.

### 2.1. Modulation of Inflammation-Related Signaling Pathways

**Figure 2:**
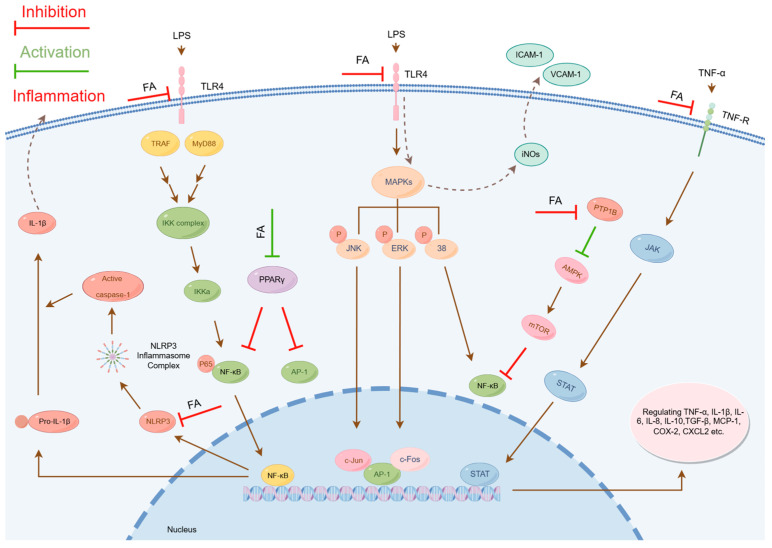
Molecular Mechanisms of the Anti-inflammatory Effects of FA. (IL-1β: Interleukin-1β; caspase-1: Cysteine-aspartic protease 1; NLRP3: NOD-like receptor protein 3; FA: Ferulic acid; LPS: Lipopolysaccharide; TLR4: Toll-like receptor 4; TRAF: Tumor necrosis factor receptor associated factor; MyD88: Myeloid differentiation primary response protein 88; IKK: IkappaB kinase; NF-κB: Nuclear factor kappa B; PPARγ: Peroxisome proliferator-activated receptor gamma; AP-1: Activator protein 1; MAPKs: Mitogen-activated protein kinases; JNK: c-Jun N-terminal kinase; ERK: Extracellular regulated kinase; c-Jun: c-Jun proto-oncogene protein; c-Fos: c-Fos proto-oncogene protein; iNOs: Inducible nitric oxide synthase; ICAM-1: Intercellular adhesion molecule 1; VCAM-1: Vascular cell adhesion molecule 1; PTP1B: Protein tyrosine phosphatase 1B; AMPK: AMP-activated protein kinase; mTOR: Mammalian target of rapamycin; TNF-α: tumor necrosis factor-α; JAK: Janus kinase; STAT: Signal transducer and activator of transcription; TGF-β: Transforming growth factor-β; MCP-1: Monocyte chemoattractant protein-1; COX-2: Cyclooxygenase-2; CXCL2: C-X-C Chemokine Ligand 2).

FA effectively modulates multiple inflammation-associated signaling pathways, including NF-κB, MAPK, NOD-like receptor protein 3 (NLRP3), JAK/STAT, peroxisome proliferator-activated receptor gamma (PPARγ), and AMP-activated protein kinase (AMPK) cascades (Figure 2). These pathways operate both independently and through crosstalk mediated by reactive oxygen species (ROS) and inflammatory cytokines, collectively constituting an intricate anti-inflammatory regulatory network.

#### 2.1.1. Inhibition of Nuclear Factor Kappa B Signaling Pathway Activation

The NF-κB signaling pathway is fundamental to cellular signal transduction. In non-activated cells, this signaling molecule associates with the inhibitory protein IκB, staying inactive within the cytoplasm. Once stimulated, IκB undergoes phosphorylation and degradation. Consequently, NF-κB is set free and migrates to the nucleus, where it triggers the transcription of target genes [64]. FA suppresses NF-κB activation through the following mechanisms:

- Inhibition of pNF-κB Phosphorylation: The phosphorylation of critical residues (e.g., Serine 536) in the p65 subunit of pNF-κB serves as a key marker of inflammatory activation. Studies have demonstrated that FA significantly reduces the phosphorylation level at this site [35,65,66,67,68,69]. This mechanism effectively prevents the nuclear translocation of NF-κB.
- Inhibition of IκBα Degradation: FA inhibits IκBα phosphorylation under inflammatory conditions, preventing its ubiquitination and degradation [59,70]. FA diminishes the entry of NF-κB into the nucleus by maintaining the stability of the IκBα-NF-κB complex.
- Inhibition of IkappaB kinase (IKK) Activity: IKK, a pivotal kinase, is essential for the phosphorylation of IκBα and the activation of the NF-κB signaling pathway. FA directly inhibits IKK activity in the cytoplasm, disrupting the IKK/IκBα phosphorylation cascade and blocking the nuclear entry of NF-κB [60].
- Inhibition of Transcriptional Activity: Within the nucleus, FA suppresses the activity of NF-κB, leading to a downregulation of proinflammatory cytokines and chemokines [61].

As a central transcriptional regulator, the extent of NF-κB inhibition directly correlates with the potency of FA’s anti-inflammatory activity. This multitiered regulatory mechanism, spanning from signal transduction to gene expression, provides a molecular rationale for the application of FA in the management of inflammation-associated diseases.

#### 2.1.2. Inhibition of Mitogen-Activated Protein Kinase Signaling Pathway Activation

The MAPK signaling pathway mediates cell surface-to-nucleus via phosphorylation cascades, ultimately regulating gene expression and cellular functions [71].

Studies have demonstrated that FA specifically inhibits phosphorylation and activation of p38 MAPK. In a lipopolysaccharide (LPS)-induced chronic constriction injury (CCI) model, FA treatment significantly decreased the expression of phosphorylated p38 MAPK in dorsal root ganglia while downregulating the production of proinflammatory cytokines, thereby effectively alleviating neuroinflammatory pain [30].

Furthermore, in diabetic nephropathy, FA simultaneously suppresses the phosphorylation of three major MAPK subtypes—p38, c-Jun N-terminal kinase (JNK), and extracellular regulated kinases 1/2 (ERK1/2)—in renal tissues, inhibiting aberrant activation of the MAPK pathway [47]. Collectively, these findings confirm that FA modulates inflammatory responses and oxidative stress by interfering with MAPK signal transduction, providing novel molecular insights for the management of diverse inflammation-associated diseases.

#### 2.1.3. Inhibition of Janus Kinase/Signal Transducer and Activator of Transcription Signaling Pathway Activation

The JAK/STAT signaling pathway, closely linked to the pathogenesis of inflammatory and autoimmune diseases such as RA [72], has emerged as a key target for FA in mediating anti-inflammatory effects. FA exerts its anti-inflammatory and immunomodulatory actions through the following mechanisms:

- Inhibition of JAK/STAT Pathway Activation: Studies reveal that in arthritis models, FA effectively suppresses aberrant activation of this pathway and reduces the expression of proinflammatory cytokines, exhibiting potent antiarthritic activity [31]. Additionally, FA shows protective effects against radiation-induced acute liver injury, which is also mediated through JAK/STAT pathway inhibition [41].
- Suppression of STAT1 Phosphorylation: In vitro experiments confirm that FA treatment inhibits LPS-stimulated STAT1 phosphorylation in BV-2 microglial cells [18]. As a pivotal transcription factor in the JAK/STAT pathway, STAT1 regulates multiple immune-inflammatory responses. By specifically blocking STAT1 activation, FA plays a crucial role in immunomodulation.

Collectively, these mechanistic insights establish a framework for developing novel therapeutic strategies targeting JAK/STAT pathway-related inflammatory disorders.

#### 2.1.4. Inhibition of NOD-like Receptor Protein 3 Inflammasome Activation

The NLRP3 inflammasome serves as a crucial sensor in the innate immune system that initiates inflammatory responses and anti-infective immunity upon detecting diverse stimuli [73]. Aberrant NLRP3 activation is strongly associated with various inflammatory and neurodegenerative disorders [74].

FA demonstrates significant neuroprotective effects by suppressing NLRP3 transcription and expression in microglia, thereby reducing the production and release of proinflammatory mediators [20,21,22]. Furthermore, FA enhances autophagic flux while downregulating NLRP3 and IL-1β expression, demonstrating renal protection in diabetic nephropathy mouse models [48]. Xiang Y. et al. reported that FA alleviates chronic respiratory depression through NLRP3 inflammasome inhibition [75]. Given the pivotal role of NLRP3 in multiple pathological processes, therapeutics targeting this inflammasome hold substantial promise for treating inflammatory and neurodegenerative diseases.

#### 2.1.5. Modulation of Peroxisome Proliferator-Activated Receptor Gamma Activity

PPARγ is a subtype of the PPAR family and functions as a ligand-activated transcription factor, exerting anti-inflammatory effects by inhibiting NF-κB signaling and the secretion of proinflammatory cytokines [76]. Research has demonstrated that FA regulates PPARγ activity through the following mechanisms:

- Upregulation of PPARγ Expression: FA enhances both gene and protein expression levels of PPARγ, thereby augmenting its activity. In a study on sodium arsenite-induced glucose intolerance and hepatotoxicity, Daryagasht M. et al. found that FA (30–100 mg/kg) upregulated hepatic PPARγ and GLUT2 protein expression in exposed mice, consequently improving glucose metabolism [77].
- Direct Binding to PPARγ: FA may function as an endogenous PPARγ ligand, directly activating PPARγ by binding to its ligand-binding domain and inducing structural changes that regulate gene expression [78]. Notably, in gentamicin-induced nephrotoxicity models, FA exhibited renal effects of protection by enhancing PPARγ gene expression and catalase (CAT) activity [49].

These findings highlight that FA, as a potential PPARγ agonist, exhibits definite anti-inflammatory capabilities, thereby laying the foundation for novel strategies in the treatment of inflammation-related diseases. Its unique PPARγ activation mechanism holds significant research value in drug development and warrants further in-depth exploration.

#### 2.1.6. Activation of the AMP-Activated Protein Kinase Signaling Pathway

As a core regulator of energy metabolism, AMPK maintains cellular energy homeostasis and modulates inflammatory signaling pathways in various pathophysiological processes [79,80]. FA exerts its dual effects through the following mechanisms targeting AMPK activation:

- Inhibition of protein tyrosine phosphatase 1B (PTP1B): PTP1B is a key phosphatase that dephosphorylates critical protein kinases. FA specifically inhibits PTP1B activity, thereby preventing AMPK dephosphorylation. Wu J. et al. demonstrated that in carbon tetrachloride-induced hepatic inflammation and fibrosis, FA directly binds to and suppresses PTP1B, promoting AMPK phosphorylation [42].
- Direct AMPK Activation: In palmitate-induced hepatocyte models of metabolic syndrome (MetS), FA activates AMPK signaling, reducing ROS levels and ameliorating oxidative stress [45]. This metabolic regulation is closely linked to its anti-inflammatory effects.

By activating AMPK, FA establishes a “metabolism-inflammation” regulatory network that confers protection in multiple inflammation-related disorders.

#### 2.1.7. Activation of the Nuclear Factor Erythroid 2-Related Factor 2 Signaling Pathway

Nuclear factor erythroid 2-related factor 2 (Nrf2), the master transcriptional regulator of cellular antioxidant defense systems, maintains redox homeostasis and modulates inflammatory responses. Emerging evidence reveals that beyond its canonical antioxidant pathway (e.g., regulating phase II detoxifying enzymes), Nrf2 exerts cytoprotective effects through epigenetic mechanisms that directly suppress proinflammatory cytokine transcription, thereby achieving synergistic antioxidant and anti-inflammatory actions [81,82]. Extensive animal studies demonstrate that nuclear factor erythroid 2-related factor 2/heme oxygenase-1 (Nrf2/HO-1) pathway activation significantly ameliorates oxidative stress and inflammatory damage in diverse pathophysiological contexts, including renal [51], hepatic [43], intestinal [56], retinal degeneration [19], and immunoregulatory disorders [83].

Mounting evidence demonstrates that FA modulates Nrf2 signaling, mediating antioxidant, anti-inflammatory, and cytoprotective effects. For instance, in LPS-induced inflammation and oxidative stress models, FA promotes Nrf2 nuclear translocation and upregulates HO-1 expression, effectively mitigating oxidative-stress-associated cellular dysfunction and apoptosis [36,62]. Furthermore, FA-induced Nrf2 activation establishes an “antioxidant–anti-inflammatory” positive feedback loop by reducing ROS accumulation to inhibit proinflammatory pathways [68].

Notably, Nrf2 does not directly suppress inflammatory mediator production. Instead, it reconstructs cellular redox homeostasis and enhances stress resistance, creating a critical microenvironmental foundation for inflammatory regulation.

#### 2.1.8. Activation of the Phosphoinositide 3-Kinase/Protein Kinase B Signaling Pathway

When extracellular stimuli activate phosphoinositide 3-kinase (PI3K), PI3K catalyzes the production of phosphatidylinositol-3,4,5-trisphosphate (PIP3) from phosphatidylinositol-4,5-bisphosphate (PIP2) at the plasma membrane. Subsequently, inactive protein kinase B (Akt) in the cytoplasm is recruited to the plasma membrane, where it interacts with PIP3, thereby fully activating Akt. Activated Akt, widespread in almost all cells and tissues, regulates numerous biological processes by modulating downstream target molecules [84].

FA exerts pleiotropic effects by regulating the PI3K/Akt signaling pathway. For example, in mice with alcoholic liver disease, FA regulates the expression of lipid-metabolism-related genes, attenuates ethanol-induced hepatic tissue injury in HepG2 cells and mice, and improves lipid metabolism disorders [44]. In palmitic-acid-treated HT22 cells, FA effectively reduced the level of oxidative stress and improved learning and memory abilities in mice, showing significant neuroprotective effects and providing a strategy for the treatment of cognitive disorders [63]. Furthermore, in a MetS model, FA (50–200 μM) significantly alleviated lipid accumulation, thereby improving insulin sensitivity and metabolic function [46].

In the inflammatory response, FA can regulate the PI3K/Akt pathway indirectly. It may modulate upstream receptor activation and influence downstream effector molecules. Meanwhile, it synergizes with antioxidant effects, regulates the release of inflammatory mediators, and cross-regulates other signaling pathways [85].

#### 2.1.9. Pathway Interaction

FA’s anti-inflammatory effects stem from the synergistic regulation of multiple pathways. Activation of the NF-κB pathway triggers an “inflammatory amplification loop” by upregulating NLRP3 transcription and downstream IL-1β/IL-18 expression; FA mitigates this process in intestinal injury models by inhibiting NF-κB activation [52,86].

Moreover, FA coordinates with various signaling pathways. In a methotrexate-induced renal injury model, it simultaneously upregulated PPARγ and Nrf2 pathways, inhibiting ROS production, blocking NF-κB/NLRP3 activation, and reducing apoptosis [50]. AMPK, integral to the inflammation–metabolism network, indirectly suppresses NF-κB by modulating fatty acid oxidation and reducing proinflammatory lipid metabolites [80]. Through the AMPK/mTOR axis, FA exhibits tissue-specific regulation: in Kawasaki disease, it activates AMPK/mTOR to inhibit NF-κB-mediated inflammation and apoptosis, while in microglia, it downregulates NLRP3 inflammasome expression to alleviate neuroinflammation [23,61].

In essence, FA orchestrates a “NF-κB/NLRP3-PPARγ/Nrf2-AMPK” network, achieving integrated effects of anti-inflammation, antioxidant defense, immunomodulation, and metabolic regulation. This multitarget approach minimizes side effects and offers a paradigm for developing natural anti-inflammatory drugs. Future research should apply systems biology methods to clarify pathway interactions and accelerate FA’s clinical translation.

### 2.2. Inhibition of Oxidative Stress

During inflammatory responses, immune cells generate excessive ROS, which when surpassing the buffering capacity of the antioxidant system, leads to cellular damage and death [87]. Critically, ROS not only directly cause oxidative damage but also act as secondary messengers to activate key inflammatory signaling pathways. Specifically, excessive ROS can trigger the NF-κB and MAPK pathways, as well as the NLRP3 inflammasome, upregulating inflammatory cytokines, thereby exacerbating inflammation and cellular damage [88,89].

**Figure 3:**
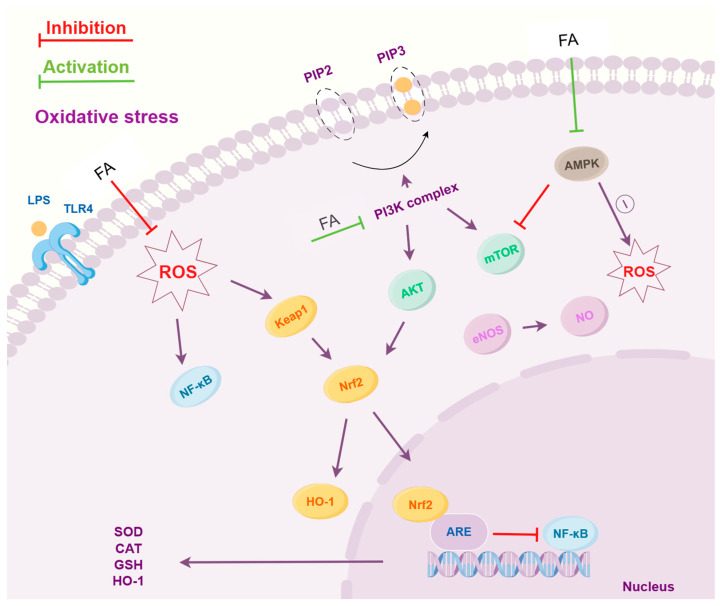
Molecular Mechanisms of the Antioxidant Effects of FA. (FA: Ferulic acid; LPS: Lipopolysaccharide; TLR4: Toll-like receptor 4; ROS: Reactive oxygen species; NF-κB: Nuclear factor kappa B; Keap1: Kelch-like ECH-associated protein 1; Nrf2: Nuclear factor erythroid 2-related factor 2; HO-1: Heme oxygenase-1; ARE: Antioxidant response element; SOD: Superoxide dismutase; CAT: Catalase; GSH: Glutathione; PIP2: Phosphatidylinositol-4,5-bisphosphate; PIP3: Phosphatidylinositol-3,4,5-trisphosphate; PI3K: Phosphoinositide 3-kinase; AKT: Protein kinase B; mTOR: Mammalian target of rapamycin; AMPK: AMP-activated protein kinase; eNOS: Endothelial nitric oxide synthase; NO: Nitric oxide).

FA’s potent antioxidant activity is attributed to its unique phenylpropanoid structure. The methoxy and phenolic hydroxyl groups on its benzene ring form stable phenoxyl radicals, enhancing its ability to scavenge ROS due to electron delocalization [90]. Consequently, FA blocks the ROS/NF-κB pathway [91] while activating Nrf2/HO-1 signaling [41], thereby ameliorating oxidative-stress-mediated cellular damage. This dual action highlights FA’s combined anti-inflammatory/antioxidant potency, providing a scientific basis for its potential application in inflammation-related diseases, as illustrated in Figure 3.

### 2.3. Regulation of Cell Adhesion Molecule Expression

CAMs are a class of membrane surface proteins that mediate interactions between cells or between cells and the extracellular matrix, promoting vascular adhesion and transendothelial migration of leukocytes, thereby amplifying inflammatory responses [92]. FA can modulate CAM expression, influencing leukocyte chemotaxis and adhesion, thereby mitigating inflammation. Studies show that FA alleviates placental inflammation in gestational diabetic rats by regulating the NF-κB pathway and downregulating ICAM-1 expression [93].

### 2.4. Immunomodulatory Effects

Complementing its direct anti-inflammatory actions, FA exerts immunomodulatory effects. FA modulates immune function through NF-κB and JAK/STAT signaling pathways, dynamic regulation of MAPK phosphorylation states, and influence on Toll-like receptor (TLR) expression [18,94,95]. This multitarget property positions FA as a promising candidate for adjunctive therapy in immunodeficiency, autoimmune disease intervention, and inflammation-related disease management.

In summary, FA exerts its anti-inflammatory effects through multiple targets and pathways. Notwithstanding these mechanisms, translational research is needed to elucidate its safety and efficacy for clinical use.

## 3. Application of Ferulic Acid in the Treatment of Excessive Inflammatory Reactions

The multimechanistic profile of FA provides a theoretical basis for its therapeutic applications across diverse inflammatory diseases. This section explores how FA’s multitarget actions translate into treatment efficacy, emphasizing disease-specific mechanisms and outcomes.

### 3.1. Role of Ferulic Acid in the Treatment of Neurodegenerative Diseases

Neurodegenerative diseases stem from dysfunctions within the nervous system. These conditions are predominantly defined by the continuous deterioration of neuronal structure and function. They include Alzheimer’s disease (AD) and Parkinson’s disease (PD). The pathogenic mechanisms that have been clarified include inflammatory responses [96,97]. Microglia and astrocytes act as crucial regulators of the inflammatory response within the central nervous system [98,99]. The activation of microglia, astrocytes. and neuroinflammation plays an important role in neurodegenerative diseases [98,100,101]. Notably, numerous studies have shown that FA confers neuroprotective effects in key brain regions, including the frontal cortex and hippocampus. These findings imply that FA holds promise as a potential therapeutic option for neurodegenerative disorders [102,103].

TLR4 mediates LPS-induced microglial activation, initiates a series of downstream signaling events, generates various cellular proinflammatory markers, and exerts harmful effects on nerve cells. Rehman S. et al. demonstrated that FA inhibits TLR4-mediated inflammatory signaling in BV-2 microglia and may inhibit NF-κB activation via a JNK-dependent mechanism [24]. Increased expression of Nurr1 promotes antineuroinflammatory responses in the brain. FA inhibits beta-amyloid (Aβ)-induced neuroinflammation in microglia by recruiting Nurr1-dependent anti-inflammatory responses in Aβ-responsive microglial cells, providing a potential alternative homeostatic shift [25]. Furthermore, FA significantly inhibits release of inflammatory factors in BV-2 microglia [26,27,28]. Similarly, in a rat model of PD induced by rotenone intoxication, FA was able to improve dyskinesia and behavioral abnormalities by restoring the balance between oxidative stress and antioxidant activity through decreasing nitric oxide (NO) and malondialdehyde (MDA) as well as increasing glutathione (GSH), in addition to exhibiting antiapoptotic properties and improving NF-κB, a key indicator of inflammation [29]. These results indicate that FA may serve as a viable therapeutic option for neurodegenerative diseases triggered by neuroinflammation.

### 3.2. Role of Ferulic Acid in the Treatment of Osteoarthrosis

#### 3.2.1. Rheumatoid Arthritis

RA is a chronic, systemic autoimmune disease primarily characterized by chronic inflammation and the progressive destruction of joints. Due to the disease’s complexity, the pathophysiological mechanisms remain incompletely understood [104]. Emerging evidence highlights the critical role of rheumatoid arthritis fibroblast-like synoviocytes (RA-FLS) in disease progression, with IL-17—a key pro-inflammatory cytokine—directly influencing the severity of FLS-mediated joint inflammation [105]. In adjuvant arthritis fibroblast-like synoviocytes (AA-FLS), FA suppresses IL-23 expression by inhibiting the IL-17/IL-17RA/STAT-3 signaling cascade, thereby attenuating IL-17-driven RA disease activity [105]. Osteoclasts, the only cells responsible for bone resorption, mediate bone erosion in various inflammatory arthritides. FA significantly reduces IL-17-induced expression of the receptor activator of nuclear factor kappa-B ligand (RANKL) and upregulates osteoprotegerin expression in AA-FLS by modulating the IL-17/IL-17RA/STAT-3 signaling pathway [32]. Similarly, Doss HM. et al. showed that FA could inhibit osteoclast differentiation and bone resorption by inhibiting the RANKL-dependent NF-κB signaling pathway [33]. Notably, there is a potential crosstalk between the liver and joints in RA patients [106]. An experiment evaluating the effects of FA on RA showed that hepatitis B virus exacerbated the progression of arthritis in mice, while paw inflammation and joint swelling were reduced after four weeks of FA administration [34]. In addition, FA inhibits the release of inflammatory factors and exhibits antiarthritic activity, which may be mediated by the inhibition of the JAK/STAT pathway [31]. Collectively, the ability of FA to contribute to the amelioration of RA is attributed to its multifaceted effects.

#### 3.2.2. Acute Gouty Arthritis

Characterized by abrupt onset and severe joint pain, acute gouty arthritis arises from sustained hyperuricemia-induced urate crystal deposition in joints and periarticular tissues, culminating in an acute inflammatory reaction [107]. While the pathogenesis involves robust activation of the NLRP3 inflammasome and NF-κB signaling, emerging evidence suggests FA may exert therapeutic effects against this condition. In a rat model of acute gouty arthritis induced by intra-articular injection of monosodium urate crystals, FA treatment attenuated paw edema by inhibiting the NF-κB pathway and downregulating NLRP3 gene transcription [35]. These outcomes imply that FA could be developed into a viable anti-inflammatory treatment.

### 3.3. Effects of Ferulic Acid on Respiratory Diseases

FA has shown potential effectiveness in numerous respiratory diseases, including acute respiratory distress syndrome (ARDS), asthma, and others. While COVID-19 has increased ARDS incidence, effective therapeutics for ARDS remain lacking [108]. Consequently, there is an urgent need to develop innovative drugs for the treatment or adjuvant therapy of ARDS. Zhang S. et al. found that FA inhibited the activation of MAPK signaling and decreased the secretion of proinflammatory factors in ARDS rats and further increased the level of IL-10 in the bronchoalveolar lavage fluid of rats, which indicated that FA had protective and anti-inflammatory effects in ARDS rats [37]. In addition, inflammation is a pivotal factor in the development of asthma, which is predominantly defined by chronic inflammation triggered by abnormal T-helper type 2 (Th2) immune reactions [109]. Thus, anti-inflammatory and bronchodilator therapies are the mainstay of asthma treatment [110]. Previous research has demonstrated that FA suppresses the allergic Th2 response and improves airway inflammation by reducing inflammatory infiltrates, lowering chemokine levels, and inhibiting cytokine levels [111]. In addition, FA showed significant antiasthmatic properties in rats suffering from allergic asthma by modulating MAPK expression [112].

### 3.4. Effects of Ferulic Acid on Cardiovascular Health

Excessive inflammatory response is a confirmed key factor in cardiovascular health [113]. Recent studies have evidenced that FA can exert protective effects against cardiovascular diseases by regulating excessive inflammation. In atherosclerosis models, FA demonstrates multiple effects: it reduces aortic plaque formation, inhibits the expression of proinflammatory cytokines, and reduces foam cell formation in plaques by inhibiting the NLRP3-IL-1β inflammatory pathway [38]. Another study [40] further reveals its cardiovascular protective mechanism: FA significantly alleviates endoplasmic reticulum stress injury in cardiomyocytes by activating the SIRT1 protein. Additionally, FA can attenuate the progression of atherosclerosis by regulating lipid metabolism and gut microbiota composition via the AMPK pathway [39]. These mechanisms collectively highlight the dual regulatory characteristics of FA on “metabolism-inflammation”—reducing lipid deposition in plaques by activating AMPK while alleviating cardiomyocyte stress injury through SIRT1 activation [39,40].

### 3.5. FA for Ulcerative Colitis

The potential of FA to enhance immune function and mitigate inflammation makes it a valuable therapeutic option for UC [114]. UC is an inflammatory bowel disease with a pathogenesis that includes a dysregulated immune response [115]. FA treats UC through multiple mechanisms, primarily by inhibiting the NF-κB signaling pathway to downregulate inflammatory gene expression, thereby preventing inflammation and histopathological injury in the colonic tissues of colitis-stricken rats [53]. Inhibition of the NLRP3 pathway: FA attenuates TNF-α induced damage in human intestinal microvascular endothelial cells (HIMECs) [54]. Reducing the release of pro-inflammatory cytokines: FA can inhibit the Interferon-gamma (IFN-γ)-induced inflammatory cascades by reducing the release of proinflammatory factors, thereby ameliorating trinitrobenzensulfonic-acid-induced inflammation [55]. Moreover, the antioxidant and antiapoptotic properties of FA provide more possibilities for the FA treatment of UC [53,54,55].

### 3.6. Therapeutic Effects of Ferulic Acid on Skin Inflammation

In atopic dermatitis, psoriasis, and other diseases of the skin system, FA shows potential therapeutic effects. The following are the mechanisms of action of FA in these diseases. Anti-inflammatory effects: FA inhibits the NF-κB signaling pathway and reduces the release of proinflammatory factors, thereby reducing skin inflammation [57]. Antioxidant effect: FA exhibits potent free radical scavenging activity, which can diminish the damage of oxidative stress on skin cells. In addition, it protects the skin from damage caused by oxidative stress by inhibiting the pro-oxidant lipoxygenase enzyme [116]. Immunomodulatory effects: In psoriasis, FA can inhibit the activation of Th1 and Th17, reduce the levels of inflammatory cytokines, and alleviate immune-mediated skin inflammation [58].

## 4. Pharmacokinetics

While FA’s therapeutic potential is evident, its clinical translation relies on understanding its pharmacokinetic properties that underpin clinical application. FA is mainly absorbed through the gastrointestinal tract [117]. However, after oral administration, it exhibits low gastrointestinal stability and poor pharmacokinetic characteristics, including low bioavailability and short plasma half-life [118,119]. Studies have shown that co-administration of FA with Honghua and clopidogrel significantly improved its pharmacokinetic parameters, suggesting that co-administration represents a promising approach to improve the bioavailability of FA [120]. Moreover, FA undergoes more efficient absorption and slower elimination in ischemic rats compared to healthy counterparts. And thus, it exhibits higher bioavailability and longer duration of action [121], suggesting that individual physiological status may influence the pharmacokinetics of FA. By comparing the pharmacokinetic behavior of FA after transdermal and intragastric administration, Yan N. et al. found that transdermal administration provides sustained drug release and avoids fluctuations in blood concentration during intragastric administration [122]. This finding suggests that different modes of administration can be selected according to therapeutic needs in clinical applications. The metabolism of FA in vivo mainly occurs in the liver, where it is converted into various metabolites via glucuronidation and sulfation [123,124], and these processes may further affect its bioavailability and efficacy. However, the academic community remains divided regarding the underlying causes of FA’s low bioavailability. Some researchers contend that FA’s limited bioavailability is primarily governed by the food matrix rather than by intestinal and hepatic metabolic processes [125]. Zeng Z. et al. demonstrated that enzymatic resistance of dietary-fiber-bound FA is a key determinant—this form of FA translocates to the colon with undigested fiber, where it undergoes microbial-mediated release and metabolism, and is ultimately excreted predominantly through feces [126]. These findings highlight the necessity to comprehensively consider the combined influence of both food matrix characteristics and metabolic pathways on FA’s bioavailability.

To improve the efficacy of FA, researchers are developing novel drug delivery systems (e.g., nanoformulations, hydrogels, microemulsions) to enhance bioavailability, stability, and absorption efficiency. For example, chitosan nanoparticles loaded with FA have a higher therapeutic index and greater thermal stability in vivo than FA and better pass the mucus barrier to enhance bioavailability [127]; synthetic hydrogels crosslinked with FA by carbomer 940 can effectively mitigate the skin damage induced by 40 Gy ^60^Co γ radiation and break through the limitations of skin penetration in conventional drug delivery [128]; and the FA self-microemulsifying drug delivery system (FA-SMEDDS) increases the area under the concentration–time curve (AUC_0−t_) in rats by 1.7-fold compared to free FA. As a novel formulation technology, the SMEDDS remarkably enhances the bioavailability and stability of FA by improving drug water solubility, optimizing intestinal absorption efficiency, and reducing the first-pass effect. Studies have shown that this system not only decreases the renal distribution and metabolism of FA but also achieves more balanced blood concentration profiles through optimized drug release kinetics [129].

Overall, the pharmacokinetic properties of FA provide an important theoretical basis for its application in the fields of anti-inflammation, antioxidation, and cardiovascular protection. However, its low bioavailability and metabolic complexity remain major challenges at present. Future technological innovations (e.g., novel delivery systems) and in-depth studies (e.g., individualized dosing) are expected to overcome these limitations and fully utilize the therapeutic potential of FA.

## 5. Toxicity and Safety

In addition to drug efficacy, toxicity is also a key factor in evaluating safety and clinical applicability. As a natural phenolic compound, FA has garnered substantial attention due to its minimal toxicity and wide-ranging biological functions. In vitro studies (refer to Table 2) show that the cytotoxic effects of FA vary across different cell lines. Overall, FA demonstrates negligible inhibitory effects and cytotoxicity toward the majority of cells (MPH, BV-2, RAW264.7, and NRK-52E cells) at concentrations ≤100 μM, indicating good safety. However, FA may exhibit cytotoxicity at higher concentrations. Notably, in certain cell types such as primary chondrocytes, FA can adversely affect cell viability even at relatively low concentrations (≥30 μM) [130]. Therefore, when using FA for experimental or therapeutic purposes, it is essential to determine appropriate concentration ranges based on specific cell types to maximize its therapeutic effects while minimizing potential cytotoxicity.

## 6. Challenges of Ferulic Acid in the Treatment of Excessive Inflammatory Response

Although FA shows significant potential in the treatment of excessive inflammation, its clinical application still faces many challenges, including

- Drug delivery and bioavailability issues: As previously mentioned, its low oral bioavailability remains a critical bottleneck.
- Balance between therapeutic dose and safety: The effective dose of FA may differ significantly from its safe dose. Prolonged high-dose intake could cause gastrointestinal discomfort, liver and kidney dysfunction, and other adverse reactions. In addition, significant interindividual metabolic differences further complicate dose adjustment and the difficulty of accurate drug administration.
- Uncertainty of therapeutic efficacy: The efficacy of FA on different types of inflammatory reactions may vary, and its mechanism of action is still incompletely elucidated. At present, most of the studies on FA are still limited to animal models and cell experiments, with insufficient large-scale and high-quality clinical research data to back it up, which brings uncertainty to its clinical application.
- Complexity of multitarget regulation: Inflammation regulation involves complex signaling networks, making it difficult for a single drug to fully cover all key targets. Although FA demonstrates potential for multitarget regulation, its specific mechanisms—particularly cross-pathway synergistic regulations—require in-depth exploration. For example, crosstalk between NF-κB and AMPK pathways requires balancing immunosuppression and metabolic regulation [45,61,131], necessitating systems biology approaches to decipher synergistic mechanisms.

In summary, although FA shows great promise in anti-inflammatory therapy, its clinical application still needs to overcome the challenges of bioavailability, dosage optimization, efficacy validation, and mechanistic studies. Future studies should focus on developing novel drug delivery systems, optimizing dosage regimens, conducting more high-quality clinical studies, and exploring the synergistic effects of FA with other anti-inflammatory drugs to fully exploit its therapeutic potential and ensure its safety.

## 7. Prospects of Ferulic Acid in the Treatment of Excessive Inflammatory Response

The multiple mechanisms of the action of FA provide a robust basis for its clinical application in inflammatory diseases. The following are possible future directions for FA in this field:

- Development of high-performance FA derivatives: Researchers can design and synthesize FA derivatives with higher purity, bioactivity, and stability via structural optimization, chemical modification, and nanotechnology. These derivatives are expected to target pleiotropic inflammation-related signaling axes concurrently, enhancing their efficacy in treating inflammatory diseases.
- Optimizing efficacy and safety: In order to maximize the therapeutic potential of FA and minimize potential adverse effects, it is critical to thoroughly investigate the optimal dosage and route of administration as well as individualized treatment regimens. In addition, exploring the efficacy differences across diverse populations will also furnish an important basis for clinical application.
- Exploring synergistic drug combinations: FA combined with other anti-inflammatory agents (e.g., immunomodulators) has shown synergistic effects. For instance, the co-administration of FA (10 mg/kg) and metformin reduces metformin’s effective dose by 75% (from 50 mg/kg to 12.5 mg/kg) and significantly mitigates the adverse effects associated with metformin monotherapy [132].
- Integration of modern science and technology: Combining modern pharmacology and biotechnology to further explore the mechanisms of action of FA in TCM formulas will forge new avenues for its modernization.

In conclusion, as mechanistic insights into FA deepen and with the rapid progress of chemical synthesis, biotechnology, and nanomedicine, the scope of FA’s potential applications in inflammatory diseases therapeutics will further expand. In the coming years, FA is expected to emerge as a pivotal natural therapeutic agent for regulating dysregulated inflammatory responses, thereby offering innovative strategies for the prevention and management of inflammation-related diseases.

## 8. Discussion

Natural products exert multipathway and multitarget mechanisms of action and are associated with fewer side effects when used in the treatment of inflammatory diseases. These attributes endow natural products and their derivatives with significant clinical potential, making them a focal point in new drug development. As a phytochemical, FA exhibits favorable anti-inflammatory effects in inflammatory diseases, highlighting its great potential as a novel anti-inflammatory agent. In this paper, we reviewed the therapeutic effects of FA and its mechanism of action in a variety of inflammatory diseases over the past decade and presented the notable observations.

First, FA exerts anti-inflammatory effects by modulating pivotal signaling pathways, thereby effectively regulating the levels of inflammatory cytokines. Second, the phenolic hydroxyl structure of FA endows it with free radical scavenging ability, which enhances antioxidant defense. Moreover, it modulates T cell polarization and TLR expression to achieve immune homeostasis regulation. Significantly, FA’s dual “anti-inflammatory and metabolic-regulatory” properties offer a safe alternative to NSAIDs for metabolic inflammation, paving the way for precision medicine approaches. FA, as a natural compound with fewer side effects, demonstrates translational potential for applications in functional foods, dietary supplements, and disease prevention and treatment drugs.

The current research on the anti-inflammatory properties of FA remains primarily at the stage of animal and cellular studies, with its clinical efficacy yet to be confirmed. To establish FA’s safety and efficacy profile, additional human clinical trials are imperative. While preclinical findings demonstrate promising potential, the clinical translation of FA faces two critical challenges: enhancing bioavailability and developing personalized dosing regimens. Future multicenter randomized controlled trials will be essential to systematically evaluate FA’s therapeutic value. Such trials would not only validate FA’s therapeutic efficacy but also bridge the translational gap between preclinical and clinical research, providing a paradigm for developing other plant-derived agents.

## Figures and Tables

**Table 1:** Mechanisms of anti-inflammatory action of FA.

Diseases	Models	Targets	Reference
Retinal Degeneration	In vitro BV-2 microglial cells vivo RD10 mice	STAT-1, TNF-α, IL-1β, NO, iNOS ↓	[18]
Retinal Degeneration	In vivo retinal degeneration pigmented rabbits	Activating Nrf2/HO-1 pathway MCP-1, IL-8, NF-κB ↓	[19]
Depression	In vivo CUMS mice	Inhibiting NF-κB pathway NLRP3, IL-1β, IL-6, TNF-α ↓	[20]
Neuroinflammation	In vitro BV-2 microglial cells	NLRP3, iNOS, NO, COX-2, ROS, IL-6, IL-1β ↓	[21]
Neuroinflammation	In vitro BV-2 microglial cells vivo brain injury mice	NLRP3, IL-1β ↓	[22]
Neuroinflammation	In vitro BV-2 microglial cells	Activating AMPK/mTOR pathway NLRP3, IL-1β, IL-6, TNF-α, ROS ↓	[23]
Neuroinflammation	In vitro BV-2 microglial cells vivo neuroinflammation mice	NF-κB, iNOS, COX-2, TNF-α, IL-1β, ROS ↓	[24]
Neuroinflammation	In vitro microglial cells	IL-10 ↑ IL-1β ↓	[25]
Neuroinflammation	In vivo neuroinflammation mice	NLRP3, IL-6, TNF-α, IL-1β ↓	[26]
Neurodegeneration	In vivo aging mice	NF-κB, IL-1β, NO ↓	[27]
AD	In vitro BV-2 microglial cells	IL-1β, IL-6, TNF-α ↓	[28]
PD	In vivo PD rats	NF-κB, NO ↓	[29]
Sciatica	In vitro GMI-R1 cells vivo CCI rats	Inhibiting RhoA/p38MAPK pathway Reduced inflammatory cell infiltration PGE2, IL-1β, IL-6, TNF-α, iNOS ↓ IL-10 ↑	[30]
Arthritic	In vivo arthritic rats	Inhibiting JAK/STAT pathway TNF-α ↓ TGF-β ↑	[31]
RA	In vitro AA-FLS and BMCs	IL-17, IL-23 ↓	[32]
RA	In vitro BMCs and RAW264.7 cells	Inhibiting NF-κB pathway	[33]
RA	In vivo CIA mice	Inhibiting NF-κB pathway	[34]
Acute Gouty Arthritis	In vivo acute gouty arthritis rats	NLRP3, NF-κB p65, TNF-α, IL-1β, NO ↓	[35]
ALI	In vitro MLE-12 cells vivo ALI mice	Activating Nrf2/HO-1 pathway	[36]
ARDS	In vivo ARDS rats	Inhibiting MAPK pathway TNF-α, IL-1β, IL-6 ↓ IL-10 ↑	[37]
AS	In vitro C3H10T1/2 cell line, RAW264.7, EA.hy926 cells vivo AS mice	NLRP3, TNF-α, IL-1β, IL-6 ↓	[38]
AS	In vivo AS mice	Activating AMPK	[39]
Cardiac Damage	In vitro H9c2 cell line and ARVM vivo cardiac dysfunction mice	Activating SIRT1	[40]
Acute Liver Injury	In vivo acute liver injury rats	Inhibiting JAK/STAT pathway Activating Nrf2 pathway ROS ↓	[41]
Liver Fibrosis	In vitro MPHs, RAW264.7 cells, LX-2 cells vivo fibrotic mice	Inhibiting NF-κB pathway Activating AMPK PTP1B, TNF-α, IL-1β ↓	[42]
Hepatic Injury	In vivo hepatic injury rats	Activating Nrf2/HO-1 pathway NF-κB, TNF-α, IL-1β ↓	[43]
ALD	In vitro HepG2 cells vivo ALD mice	Activating AMPK and PI3K/AKT pathway	[44]
MetS	In vitro HepG2 cells	Activating AMPK pathway	[45]
MetS	In vitro HepG2 cells	Activating PI3K/AKT pathway PPARγ ↑	[46]
DN	In vitro NRK-52E cells vivo DN rats	Inhibiting MAPK and NF-κB pathway ROS, NO, IL-1β, IL-6, TNF-α, COX-2, iNOS↓	[47]
DN	In vivo DN mice	NLRP3, TNF-α ↓	[48]
Nephrotoxicity	In vivo nephrotoxicity rats	PPARγ ↑	[49]
Nephrotoxicity	In vivo nephrotoxicity rats	Activating Nrf2/ARE/HO-1 pathway NF-κB, NLRP3, ROS ↓ PPARγ ↑	[50]
AKI	In vivo AKI mice	Inhibiting NF-κB pathway Activating Nrf2/HO-1 pathway TNF-α, IL-1β, iNOS, COX-2 ↓ Reduced inflammatory cell infiltration	[51]
Intestinal Injury	In vivo intestinal injury mice	NF-κB NLRP3 IL-18 IL-1β ↓	[52]
UC	In vivo UC rats	Inhibiting NF-κB pathway iNOS NO ↓	[53]
UC	In vitro HIMECs vivo UC rats	NLRP3 IL-6 IL-12 IL-1β ↓	[54]
UC	In vivo UC rats	TNF-α IL-1β IL-6 COX-2 iNOs ↓	[55]
Intestinal Epithelial Barrier Dysfunction	In vitro IEC-6 cells	Activating Nrf2/HO-1 pathway ROS, NO ↓	[56]
Atopic Dermatitis	In vitro THP-1 cells vivo atopic dermatitis mice	Inhibiting NF-κB pathway IgE TNF-α IL-6 ↓	[57]
Psoriasis	In vivo psoriasis-like skin injury mice	IL-23 IL-1β ↓	[58]
Endometritis	In vitro BEECs	Inhibiting NF-κB and MAPK pathway IL-1β, IL-6, TNF-α, IL-8 ↓	[59]
Inflammation	In vitro 3T3-L1 adipocytes and RAW264.7 cells	Inhibiting JNK/ERK and NF-κB pathway TNF-α, IL-6, IL-1β, MCP-1↓	[60]
KD	In vitro HUVECs vivo KD mice	Activating AMPK/mTOR pathway Inhibiting NF-κB pathway IL-1β, IL-6, TNF-α, CXCL10 ↓	[61]
Mastitis	In vitro BMECs	Activating Nrf2 IL-1β, IL-6, TNF-α, ROS, COX-2, NF-κB ↓	[62]
Cognitive Impairment	In vitro HT22 cells vivo cognitive impairment mice	Activating IRS1/PI3K/AKT/GSK-3β pathway	[63]

(Footnote: ↓ indicates downregulation of expression, ↑ indicates upregulation of expression).

**Table 2:** Cytotoxic effects of FA across different cell lines.

Diseases	Models	Concentration	Cytotoxicity	Assay	Reference
Neuroinflammation	BV-2 microglial cells	19, 38, 76, 152 μM	Nontoxicity	CCK-8	[21]
Neuroinflammation	BV-2 microglial cells	2.5, 5, 10 μM	Nontoxicity	CCK-8	[22]
Neuroinflammation	BV-2 microglial cells	40, 80, 160μM	Nontoxicity	MTT	[23]
Neuroinflammation	BV-2 microglial cells	10, 100 μM	Not mentioned	__	[24]
AD	BV-2 microglial cells	55 μM	Nontoxicity	MTT	[28]
Sciatica	GMI-R1 cells	2 μM	Nontoxicity	CCK-8	[30]
RA	AA-FLS	25, 50, 100 μM	≥100 μM	MTT	[32]
RA	RAW264.7 cells	25, 50, 100 μM	≥100 μM	MTT	[33]
Osteoarthritis	Primary chondrocytes patients	5, 10 μM	≥30 μM	CCK-8	[130]
ALI	MLE-12 cells	0.1 μM	Not mentioned	__	[36]
Liver Fibrosis	MPHs	25 μM	≥100 μM	CCK-8	[42]
	RAW264.7 cells	100 μM	Nontoxicity	CCK-8	
	LX-2 cells	25 μM	≥50 μM	CCK-8	
ALD	HepG2 cells	50, 100 μM	≥200 μM	MTT	[44]
MetS	HepG2 cells	50, 100, 200 μM	Not mentioned	__	[45]
MetS	HepG2 cells	50, 100, 200 μM	≥1 mM	methylene blue	[46]
DN	NRK-52E cells	75 μM	≥100 μM	MTT	[47]
UC	HIMECs	125, 250, 500 μM	Not mentioned	__	[54]
Intestinal Epithelial Barrier Dysfunction	IEC-6 cells	5, 10, 20 μM	Not mentioned	__	[56]
Cardiac Damage	H9c2 cell line	5 μM	Nontoxicity	FDA	[40]
Atopic Dermatitis	THP-1 cells	5, 10 μM	Nontoxicity	TUNEL	[57]
Endometritis	BEECs	40, 80, 120 μM	Nontoxicity	MTT	[59]
Inflammation	3T3-L1 adipocytes	1, 10, 50 μM	Nontoxicity	MTT	[60]
KD	HUVECs	20 μM	Nontoxicity	CCK-8	[61]
Cognitive Impairment	HT22 cells	150, 300, 600 μM	Nontoxicity	CCK-8	[63]

## Data Availability

No new data were created or analyzed in this study. Data sharing is not applicable to this article.

## Abbreviations

FA	Ferulic acid
RA	Rheumatoid arthritis
UC	Ulcerative colitis
NF-κB	Nuclear factor kappa B
MAPK	Mitogen-activated protein kinase
JAK/STAT	Janus kinase/signal transducer and activator of transcription
NSAIDs	Nonsteroidal anti-inflammatory drugs
TCMs	Traditional Chinese medicines
TNF-α	Tumor necrosis factor-α
IL-1β	Interleukin-1β
CAM	Cell adhesion molecule
NLRP3	NOD-like receptor protein 3
AMPK	AMP-activated protein kinase
PPARγ	Peroxisome proliferator-activated receptor gamma
ROS	Reactive oxygen species
IKK	IkappaB kinase
LPS	Lipopolysaccharide
CCI	Chronic constriction injury
JNK	c-Jun N-terminal kinase
ERK	Extracellular regulated kinase
CAT	Catalase
PTP1B	Protein tyrosine phosphatase 1B
MetS	Metabolic syndrome
Nrf2/HO-1	Nuclear factor erythroid 2-related factor 2/heme oxygenase-1
PI3K/Akt	Phosphoinositide 3-kinase/protein kinase B
PIP3	Phosphatidylinositol-3,4,5-trisphosphate
PIP2	Phosphatidylinositol-4,5-bisphosphate
TLR	Toll-like receptor
AD	Alzheimer’s disease
PD	Parkinson’s disease
NO	Nitric oxide
MDA	Malondialdehyde
GSH	Glutathione
RA-FLS	Rheumatoid arthritis fibroblast-like synoviocytes
AA-FLS	Adjuvant arthritis fibroblast-like synoviocytes
RANKL	Receptor activator of nuclear factor kappa-B ligand
ARDS	Acute respiratory distress syndrome
Th2	T-helper type 2
HIMECs	Human intestinal microvascular endothelial cells
IFN-γ	Interferon-gamma
iNOs	Inducible nitric oxide synthase
MCP-1	Monocyte chemoattractant protein-1
CUMS	Chronic unpredictable mild stress
COX-2	Cyclooxygenase-2
PGE2	Prostaglandin E2
CIA	Collagen-induced arthritis
ALI	Acute lung injury
AS	Atherosclerosis
ARVM	Adult rat ventricular myocytes
ALD	Alcoholic liver disease
DN	Diabetic nephropathy
AKI	Acute kidney injury
IgE	Immunoglobulin E
KD	Kawasaki disease
CXCL	C-X-C Chemokine Ligand
BMECs	Bovine mammary epithelial cells
SMEDDS	Self-microemulsifying drug delivery system
AUC_0−t_	Area under the concentration–time curve
